# Transcriptomic Responses of the Marine Diatom *Phaeodactylum tricornutum* to High Carbon and Low Nitrogen Stress

**DOI:** 10.1002/ece3.72754

**Published:** 2026-01-09

**Authors:** Yi Zhang, Jiawen Duan, Yimeng Zheng, Xiaoqi Chen, Chenhui Li, Zhenyu Xie, Aiyou Huang

**Affiliations:** ^1^ State Key Laboratory of Marine Resource Utilization in the South China Sea Hainan University Haikou Hainan Province China; ^2^ School of Marine Biology and Fisheries Hainan University Haikou Hainan China; ^3^ Laboratory of Development and Utilization of Marine Microbial Resource Hainan University Haikou Hainan Province China; ^4^ Key Laboratory of Tropical Hydrobiology and Biotechnology of Hainan Province Haikou Hainan China; ^5^ Wenchang Advanced Fisheries Research Institute Hainan University Wenchang Hainan China

**Keywords:** carbon enrichment, carbon‐nitrogen balance, diatom adaptation, nitrogen limitation, *Phaeodactylum tricornutum*, transcriptomics

## Abstract

Diatoms play a pivotal role in global biogeochemical cycling and marine primary productivity, making them ideal model organisms for understanding how phytoplankton respond to environmental fluctuations associated with global climate change. In natural marine systems, diatoms frequently encounter simultaneous variations in carbon and nitrogen availability, yet most previous studies have examined the effects of these factors in isolation. To elucidate the integrated transcriptional mechanisms underlying diatom acclimation to coupled carbon–nitrogen (C—N) imbalance, we employed RNA sequencing (RNA‐Seq) to characterize the global transcriptional response of the model diatom *Phaeodactylum tricornutum* to high CO_2_ (~2000 μatm) and low nitrogen (10% of nitrogen concentration in f/2 medium) under parallel culture conditions. The results revealed both shared and distinct transcriptional responses between the two treatments. Key genes involved in carbon metabolism, such as phosphoglycerate mutase (PGAM_7) and dihydrolipoamide succinyltransferase (PHATRDRAFT_40430), were significantly upregulated, indicating enhanced glycolytic and TCA cycle activity. In contrast, the Calvin‐cycle enzyme fructose‐1,6‐bisphosphatase (FBPC4) was downregulated. Genes associated with nitrogen assimilation‐including nitrate reductase (PHATRDRAFT_54983), nitrite reductases (PHATRDRAFT_13154, PHATRDRAFT_8155), and ferredoxin–nitrite reductase (PHATRDRAFT_27757)‐were strongly induced under both conditions. Pathway enrichment analysis further indicated the activation of lactic acid fermentation and nitrogen salvage pathways, suggesting a metabolic shift toward energy conservation and nutrient recycling. Collectively, these findings provide an overview of the transcriptional adjustments that enable *P. tricornutum* to maintain C—N homeostasis under high CO_2_ and low nitrogen stress, offering new insights into diatom metabolic plasticity under changing ocean conditions.

## Graphical abstract

To elucidate the integrated transcriptional mechanisms underlying diatom acclimation to coupled carbon–nitrogen (C—N) imbalance, we employed RNA sequencing (RNA‐Seq) to characterize the global transcriptional response of the model diatom *Phaeodactylum tricornutum* to high CO2 (~2000 μatm) and low nitrogen (10% of nitrogen concentration in f/2 medium) under parallel culture conditions. The results revealed both shared and distinct transcriptional responses between the two treatments. The findings provide an overview of the transcriptional adjustments that enable *P. tricornutum* to maintain C—N homeostasis under high CO_2_ and low nitrogen stress, offering new insights into diatom metabolic plasticity under changing ocean conditions.

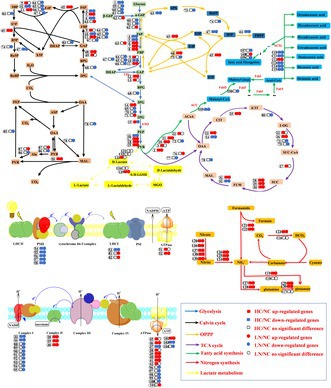

## 1 Introduction

Diatoms are crucial marine primary producers and a key contributor to ocean biomass (Nelson et al. 1995; Raven and Waite 2004; Zaslavskaia et al. 2000). Often referred to as the “grasslands of the ocean,” diatoms play a vital role in marine food webs due to their abundance, diversity, and high photosynthetic efficiency. As major carbon fixers, they contribute substantially to the global biogeochemical cycles of carbon and nitrogen and serve as an essential model for understanding biological responses to global climate change and anthropogenic pollution.

*Phaeodactylum tricornutum* , a model marine diatom, has been extensively used to study diatom physiology, metabolism, and gene regulation (Bowler et al. 2008; Siaut et al. 2007; De Risco et al. 2009). Its rapid growth, efficient carbon fixation, and ability to synthesize valuable compounds‐such as polyunsaturated fatty acids, pigments, and proteins‐make it important for both basic research and biotechnological applications (Hu et al. 2008; Stukenberg et al. 2018).

In natural marine environments, diatoms frequently experience large fluctuations in carbon and nitrogen availability. Elevated partial pressure of CO_2_ (pCO_2_) can occur in coastal upwelling zones and eutrophic waters, sometimes exceeding 1000 μatm (Feely et al. 2018; Sunda and Cai 2012), while dissolved inorganic nitrogen varies from replete in coastal regions to severely depleted in oligotrophic oceans (Gruber 2008). Such variations impose strong selective pressure for metabolic flexibility.

Previous studies have shown that elevated CO_2_ (~2000 μatm) can enhance carbon fixation, lipid accumulation, and photosynthetic electron transport in *P. tricornutum* , accompanied by increased NADPH supply and upregulation of key enzymes in the Calvin cycle and pentose phosphate pathway (Wu et al. 2019, 2015; Li et al. 2012). Such metabolic enhancement suggests that *P. tricornutum* readjusts its central carbon metabolism under high CO_2_, redistributing energy and carbon skeletons to support accelerated growth and lipid biosynthesis. Similarly, structural and physiological studies in diatoms have demonstrated that elevated CO_2_ can alter pyrenoid organization and carbon fixation efficiency (Shimakawa et al. 2024), further indicating that high‐CO_2_ exposure reshapes diatom photosynthetic and metabolic networks.

Conversely, nitrogen limitation generally results in reduced growth and photosynthetic efficiency but promotes lipid accumulation and pigment degradation (Alipanah et al. 2015; Levitan et al. 2015; Yang et al. 2014). Multi‐omics analyses have revealed that under nitrogen deficiency, *P. tricornutum* reallocates nitrogen from proteins and chlorophyll to lipid synthesis and stress‐response pathways (Remmers et al. 2018). Recent comparative studies have extended these findings across diatom taxa. For example, *Chaetoceros socialis* and *Thalassiosira* spp. display both conserved and species‐specific transcriptional adjustments under nitrogen limitation, including altered nitrate transporter (NRT) expression, amino acid metabolism, and lipid biosynthesis (Pelusi et al. 2023; Ma et al. 2024). Likewise, *P. tricornutum* exhibits extensive transcriptional reprogramming during nitrogen deprivation (Scarsini et al. 2022), highlighting the plasticity of its metabolic regulation. Broader analyses integrating multiple stressors have further shown that *Thalassiosira* species and *P. tricornutum* share a core environmental stress response involving redox balance and central carbon metabolism, while differing in regulatory specificity (Li et al. 2021, 2023). In addition to these metabolic adjustments, transcriptional regulation plays a pivotal role in diatom acclimation. For instance, the MYB transcription factor family in *P. tricornutum* responds strongly to nitrogen deficiency and diurnal cycles (Wang et al. 2022), underscoring the central role of gene regulatory networks in coordinating nutrient and energy metabolism.

Nevertheless, because previous investigations have primarily examined the effects of either elevated CO_2_ or nitrogen limitation in isolation, our understanding of the integrated transcriptional regulation governing carbon–nitrogen metabolic coupling remains limited.

Therefore, in this study, we conducted a parallel transcriptomic analysis of *P. tricornutum* under two ecologically relevant stresses: high carbon (HC, ~2000 μatm pCO_2_) and low nitrogen (LN, one‐tenth of normal nitrate). By comparing these treatments, we aimed to identify both common and stress‐specific transcriptional responses involved in maintaining metabolic homeostasis under C—N imbalance. Our findings provide transcriptome‐wide insights into diatom acclimation strategies and lay a theoretical foundation for understanding the coordination between carbon and nitrogen metabolism in marine microalgae.

## 2 Materials and Methods

### 2.1 Culture Conditions

The alga *P. tricornutum* Bohlin was sourced from the Microalgae Culture Centre (MACC) at Ocean University of China. The culture of algal cells was conducted in a NaHCO_3_‐free, sterilized artificial seawater mixed with the f/2 nutritional supplement (Guillard 1975). Cultures were subjected to incubation under cool white fluorescent illumination (~100 μmol m^−2^ s^−1^) at a temperature of 20°C, adhering to a 12‐h dark to 12‐h light photoperiod, and were inoculated at an initial density corresponding to a cell density of 0.05 at OD_730nm_. The cell proliferation was tracked by employing a UV/visible spectrophotometer (model UV‐1800, Shimadzu, Japan) to assess the absorbance at 730 nm (expressed as A_730nm_).

For the high CO_2_ (HC) treatment, cultures were aerated with a mixture of air and pure CO_2_ to achieve a target gas‐phase partial pressure of ~2000 μatm, whereas the normal CO_2_ (NC) and low nitrogen (LN) treatments were aerated with ambient air (~400 μatm pCO_2_). The aeration rate was maintained at 500 mL min^−1^. The chosen CO_2_ level follows previous diatom studies that simulated future ocean acidification scenarios using 1000–2000 μatm CO_2_ (Wu et al. 2010; Li et al. 2012; Xu et al. 2021).

For the low nitrogen treatment, f/2 medium was prepared with one‐tenth of the normal NaNO₃ concentration (7.5 mg L^−1^ ~ 88 μM), based on reported nitrate uptake thresholds for *P. tricornutum* (Huang et al. 2019; Feely et al. 2023). The high CO_2_ and normal treatments received full‐strength f/2 medium. Each treatment was implemented in three parallel replicates. Following an incubation period of 7 days, when cultures were in the exponential growth phase as determined by preliminary growth curve measurements, cell pellets were collected and rinsed with distilled water at 5000 g for 4 min. The pellets were frozen in liquid nitrogen and stored at −80°C.

### 2.2 RNA Extraction

Total RNA was extracted from cell pellets using TRIzol Reagent (Plant RNA Purification Reagent for plant tissue; Invitrogen) according to the manufacturer's instructions and genomic DNA was removed using DNase I (TaKara). Then RNA quality was determined by 2100 Bioanalyser (Agilent) and quantified using the NanoDrop ND‐2000 spectrophotometer (Thermo Fisher Scientific). Only high‐quality RNA samples (OD260/280 = 1.8 ~ 2.2, OD260/230 ≥ 2.0, RIN ≥ 6.5, 28S:18S ≥ 1.0, > 2 μg) were used to construct sequencing library.

### 2.3 Library Preparation and Sequencing

RNA‐seq transcriptome library was prepared following the TruSeq RNA Sample Preparation Kit from Illumina (San Diego, CA, USA) following the manufacturer's instructions, with 1 μg of total RNA as input. Shortly, messenger RNA was isolated according to the poly(A) selection method by oligo (dT) beads and then fragmented by fragmentation buffer first. Secondly, double‐stranded cDNA was synthesized using a SuperScript Double‐Stranded cDNA Synthesis Kit (Invitrogen, CA, USA) with random hexamer primers (Illumina). Then the synthesized cDNA was subjected to end‐repair, phosphorylation, and “A” base addition according to Illumina's library construction protocol. Libraries were size selected for cDNA target fragments of 200–300 bp on 2% Low Range Ultra Agarose, followed by PCR amplification using Phusion DNA polymerase (NEB, USA) for 15 PCR cycles. After quantification by TBS380, paired‐end RNA‐seq sequencing library was sequenced with the Illumina HiSeq X Ten/NovaSeq 6000 sequencer (2 × 150 bp read length).

### 2.4 Read Mapping and Quantification

The raw paired end reads were trimmed and quality controlled by SeqPrep (v1.2; https://github.com/jstjohn/SeqPrep) (John 2013) and Sickle (v1.33; https://github.com/najoshi/sickle) (Joshi and Fass 2011) with default parameters. Clean reads were separately aligned to the reference genome in orientation mode using TopHat (v2.0.0; http://tophat.cbcb.umd.edu/) (Trapnell et al. 2009) with Bowtie2 (v2.2.9). Mapping allowed up to two mismatches without insertions or deletions. Gene expression levels were quantified using RSEM (v1.3.1; http://deweylab.biostat.wisc.edu/rsem/) (Li and Dewey 2011). Raw expected counts from RSEM were used for differential expression analysis, while fragments per kilobase of transcript per million mapped reads (FPKM) values were used for expression visualization.

### 2.5 Differential Expression and Functional Enrichment Analysis

Differentially expressed genes (DEGs) between two different treatments were identified using the EdgeR package (v3.12.0; http://www.bioconductor.org/packages/2.12/bioc/html/edgeR.html) (Robinson et al. 2010) (Empirical analysis of Digital Gene Expression in R). Prior to analysis, genes with very low expression (counts per million < 1 in fewer than two samples) were filtered out. Library sizes were normalized using the trimmed mean of *M*‐values (TMM) method. Differential expression was assessed using a negative binomial generalized linear model, and statistical significance was determined using the Benjamini‐Hochberg false discovery rate (FDR) correction.

Genes with |log_2_FC| ≥ 1 and FDR < 0.05 were considered significantly differentially expressed. Volcano plots were generated to visualize the distribution of DEGs, with log_2_ fold change (log_2_FC) on the *x*‐axis and –log_10_ adjusted *p*‐value on the *y*‐axis. Hierarchical clustering heatmaps were constructed using the top 50 DEGs (ranked by |log_2_FC|) in each comparison (NC vs. LN and NC vs. HC). Both volcano plots and heatmaps were generated using the Majorbio Cloud Platform (https://www.majorbio.com/). The identified DEGs were subjected to functional enrichment analysis, including Gene Ontology (GO) enrichment using GOATOOLS (v1.0.6; https://github.com/tanghaibao/Goatools) (Klopfenstein et al. 2018) and Kyoto Encyclopedia of Genes and Genomes (KEGG) pathways enrichment using KOBAS (v3.0.3; http://kobas.cbi.pku.edu.cn/home.do) (Xie et al. 2011).

### 2.6 Quantitative Real‐Time PCR

Total RNA was extracted as described in Section 2.2. The HiScript Q RT SuperMix for qPCR with gDNA wiper (Vazyme, Nanjing, China) was used to generate cDNA according to the user's manual. All procedures were performed on ice. Primers (Table S1) were designed according to randomly selected transcriptomic sequenced genes. Transcript levels were quantified using reagents from the ChamQ SYBR Color qPCR Master Mix (Vazyme, Nanjing, China) and an ABI7500 Real‐Time PCR Reaction system (Applied Biosystems, USA). The cycling parameters for quantitative real‐time PCR (qPCR) were 95°C for 5 min, followed by 40 cycles at 95°C for 5 s, 55°C for 30 s, and 72°C for 40 s. Melting curve analyses were performed to ensure that only a single specific DNA fragment was amplified.

Gene expression levels were normalized against two internal reference genes (RPS and 18S), and relative expression was calculated using the 2^−ΔΔCt^ method (Livak and Schmittgen 2001). Each treatment included three independent biological replicates.

### 2.7 Statistical Analysis

Statistical analyses of transcriptome data were performed using R packages including edgeR and GOATOOLS. Differential expression was assessed using a negative binomial generalized linear model, and significance was determined by the Wald test with Benjamini–Hochberg FDR correction (FDR < 0.05). GO and KEGG enrichment analyses were conducted using hypergeometric tests followed by FDR correction to identify significantly overrepresented functional categories. Pearson correlation analysis and principal component analysis (PCA) were employed to evaluate the consistency among biological replicates and the overall transcriptional variation among treatments. For qPCR data, statistical differences were determined using two‐tailed Student's t‐tests and one‐way ANOVA, with *p* < 0.05 considered statistically significant. All analyses were performed using data from three independent biological replicates for each treatment condition (NC, HC, and LN).

## 3 Results

### 3.1 Sequencing Quality Assessment

Comprehensive statistical analyses were performed on both raw and quality‐controlled sequencing data, with the outcomes displayed in Table S2. Each sample generated an average of 54,676,132 clean reads. The Q20 (the percentage of bases in the sequence with a quality score above 99%) and Q30 (the percentage of bases in the sequence with a quality score above 99.9%) values of the sequencing data ranged from 98.58% to 98.72% and 95.41% to 95.81%, respectively. These high‐quality sequencing data provide a robust foundation for subsequent analyses.

We aligned the high‐quality sequences that have undergone quality control with the reference genome. The mapping rates of each sample's clean data to the designated reference genome ranged from 90.68% to 92.74%, indicating high data accuracy and low presence of contaminating DNA (Table S3). The amount of uniquely mapped reads is also an important metric, as it indicates the proportion of reads that map to a unique location in the reference genome. A high percentage of uniquely mapped reads (greater than 70%) is desirable, reducing the possibility of mapping errors or ambiguous mapping locations (Conesa et al. 2016). In our setting, and the percentage of uniquely mapped reads to a single location on the reference genome accounted for 88.13% to 90.11% of the clean reads. This indicates that the selected reference genome is appropriate, there is no contamination in the relevant experiments, and the sequencing results meet the requirements for subsequent analysis.

### 3.2 Correlation Analysis of Gene Expression Between Groups

**FIGURE 1:**
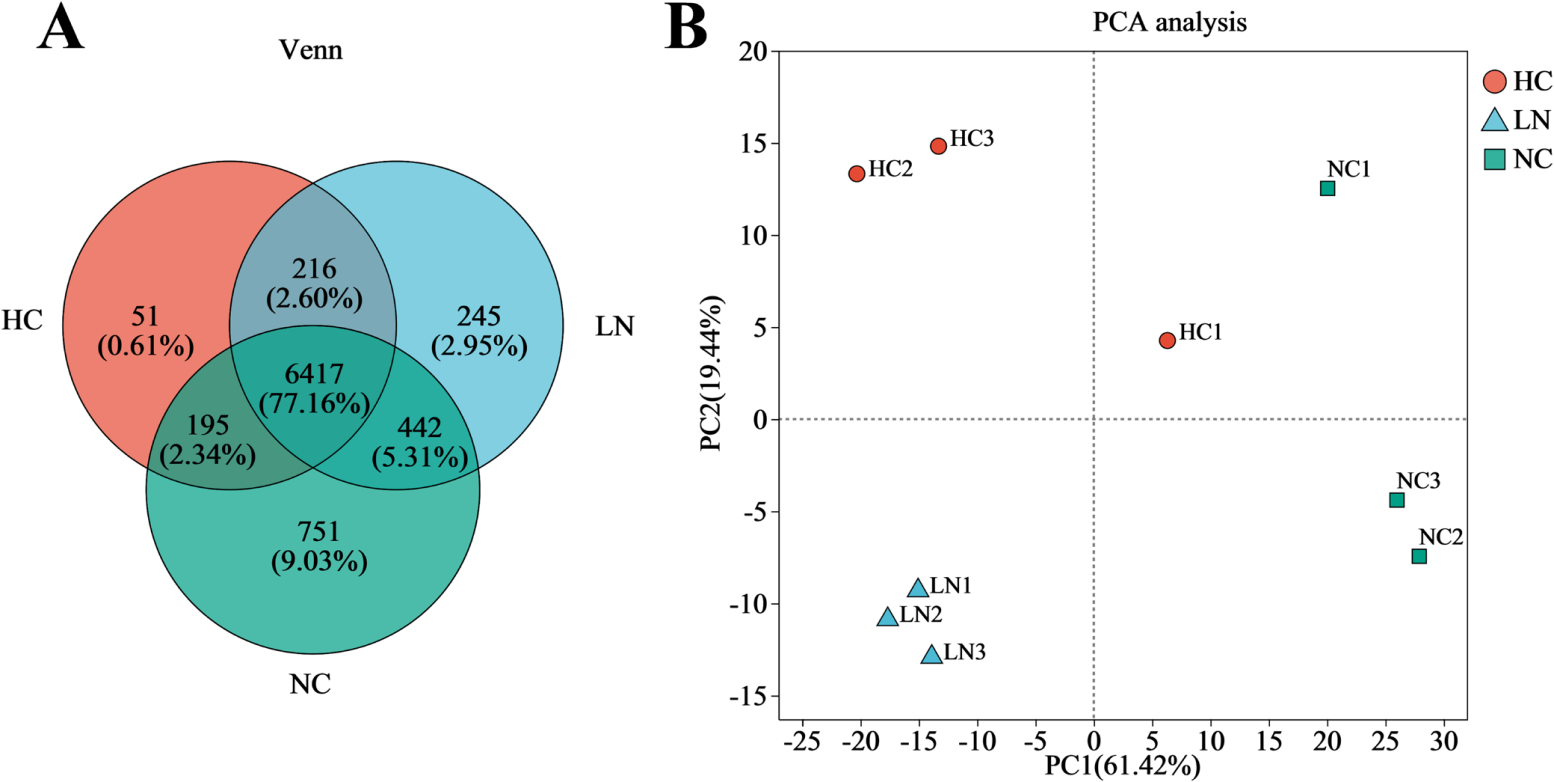
Correlation analysis of gene expression between three experimental groups. (A) Venn diagram illustrating the commonly and uniquely expressed genes in the high CO_2_ conditions (HC), in the low nitrogen conditions (LN), and in the normal conditions (NC). (B) The PCA based on all gene expression levels illustrates the correlation of gene expression between groups. The values in parentheses on the axes represent the percentage of total variance explained by each principal component. Samples from the same group are indicated by the same color and shape, with red circles representing the high CO_2_ conditions, blue triangles representing the low nitrogen conditions, and green diamonds representing the normal conditions.

To compare the gene expression profiles among treatments, we performed Venn analysis based on expressed genes. Expressed genes were defined as those with fragments per kilobase of transcript per million mapped reads (FPKM) ≥ 1 in at least one condition. Venn analysis was performed to compare the expressed genes among low nitrogen conditions (LN), high CO_2_ conditions (HC), and normal conditions (NC), in order to assess the overlap of actively transcribed genes among treatments. A total of 6417 genes were commonly expressed across all three groups, while 51, 751, and 245 genes were uniquely expressed in high CO_2_ conditions, normal conditions, and low nitrogen conditions, respectively. Between high CO_2_ conditions and normal conditions, 6612 genes were expressed in both groups, with 267 genes uniquely expressed in high CO_2_ conditions and 1193 genes uniquely expressed in normal conditions. Between low nitrogen conditions and normal conditions, 6859 genes were expressed in both groups, with 461 genes uniquely expressed in low nitrogen conditions and 946 genes uniquely expressed in normal conditions (Figure 1A). For context, the *P. tricornutum* genome contains ~12,233 protein‐coding genes, of which ~9000–10,000 were detected as expressed under our experimental conditions. PCA analysis reflected distinct clustering of replicate samples within the same experimental group and clear divergence across different groups. The first (PC1) and second (PC2) principal components accounted for 61.42% and 19.44% of the variation, respectively (Figure 1B).

### 3.3 Differential Expression of mRNAs Under Low Nitrogen and High CO_2_ Conditions

#### 3.3.1 Identification of Differentially Expressed Genes (DEGs)

**FIGURE 2:**
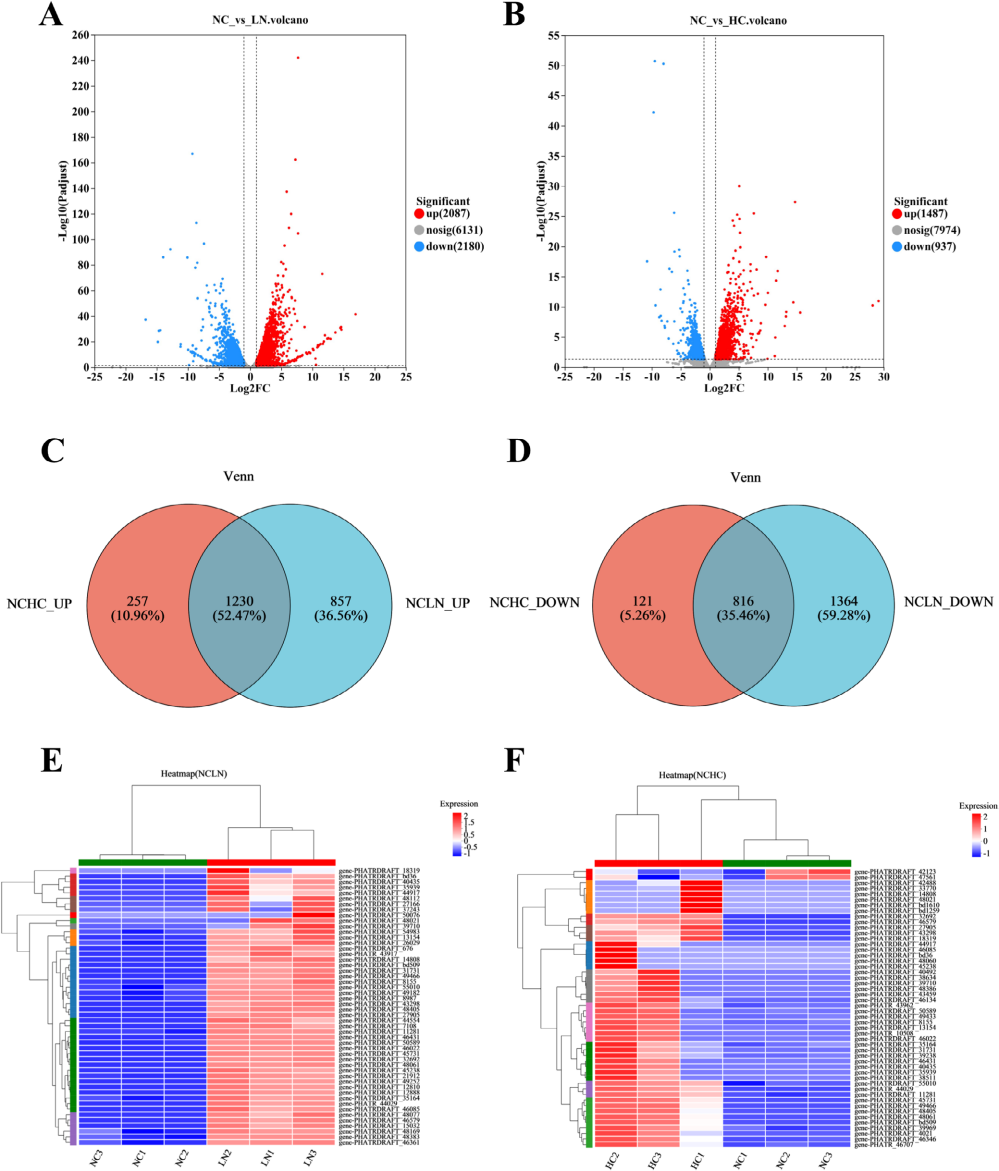
Differential expression analysis of genes under low nitrogen (LN) and high CO_2_ (HC) conditions. (A, B) Volcano plots of differentially expressed genes. (A) NC vs LN; (B) NC vs HC. In the volcano plot, each dot represents a gene: Red dots signify upregulated genes, blue dots signify downregulated genes, and black dots indicate genes that are not significantly different. (C, D) Overlap of differentially expressed genes (DEGs) under high CO_2_ (HC) and low nitrogen (LN) conditions. (C) Upregulated genes. (D) Downregulated genes. The overlapping regions indicate shared transcriptional responses between both stress treatments. (E, F) Hierarchical clustering heatmaps of differentially expressed genes (DEGs). (E) Heatmap of the top 50 DEGs (ranked by |log_2_FC|) in the NC vs. LN comparison. (F) Heatmap of the top 50 DEGs in the NC vs. HC comparison. Expression values were normalized by *Z*‐score transformation. Each row represents one gene, and each column corresponds to a biological replicate. The color scale indicates relative expression levels, with red denoting upregulation and blue denoting downregulation.

Gene expression transcriptome responses under low nitrogen and high CO_2_ conditions were analyzed using high‐throughput RNA sequencing. A total of 10,398 genes were detected, among which 4632 were significantly differentially expressed (|log_2_FC| ≥ 1 and FDR < 0.05). Under low nitrogen conditions, 4267 DEGs were identified, comprising 2087 upregulated and 2180 downregulated. Under high CO_2_ conditions, 2424 DEGs were identified, comprising 1487 upregulated and 937 downregulated genes (Figure 2A,B, Tables S4 and S5). These results indicate pronounced transcriptional reprogramming under both stress conditions, with low nitrogen conditions eliciting a broader transcriptomic response than high CO_2_ conditions.

The Venn diagram analysis revealed substantial overlap in transcriptional responses between the two stress conditions. Specifically, 1230 genes were commonly upregulated under both high CO_2_ and low nitrogen treatments, whereas 257 and 857 genes were uniquely upregulated under high CO_2_ and low nitrogen, respectively (Figure 2C). Likewise, 816 genes were downregulated in both treatments, with 121 and 1364 genes showing condition‐specific downregulation under high CO_2_ and low nitrogen, respectively (Figure 2D). These results suggest that *P. tricornutum* exhibits both shared and distinct transcriptional strategies in response to carbon and nitrogen perturbations.

**FIGURE 3:**
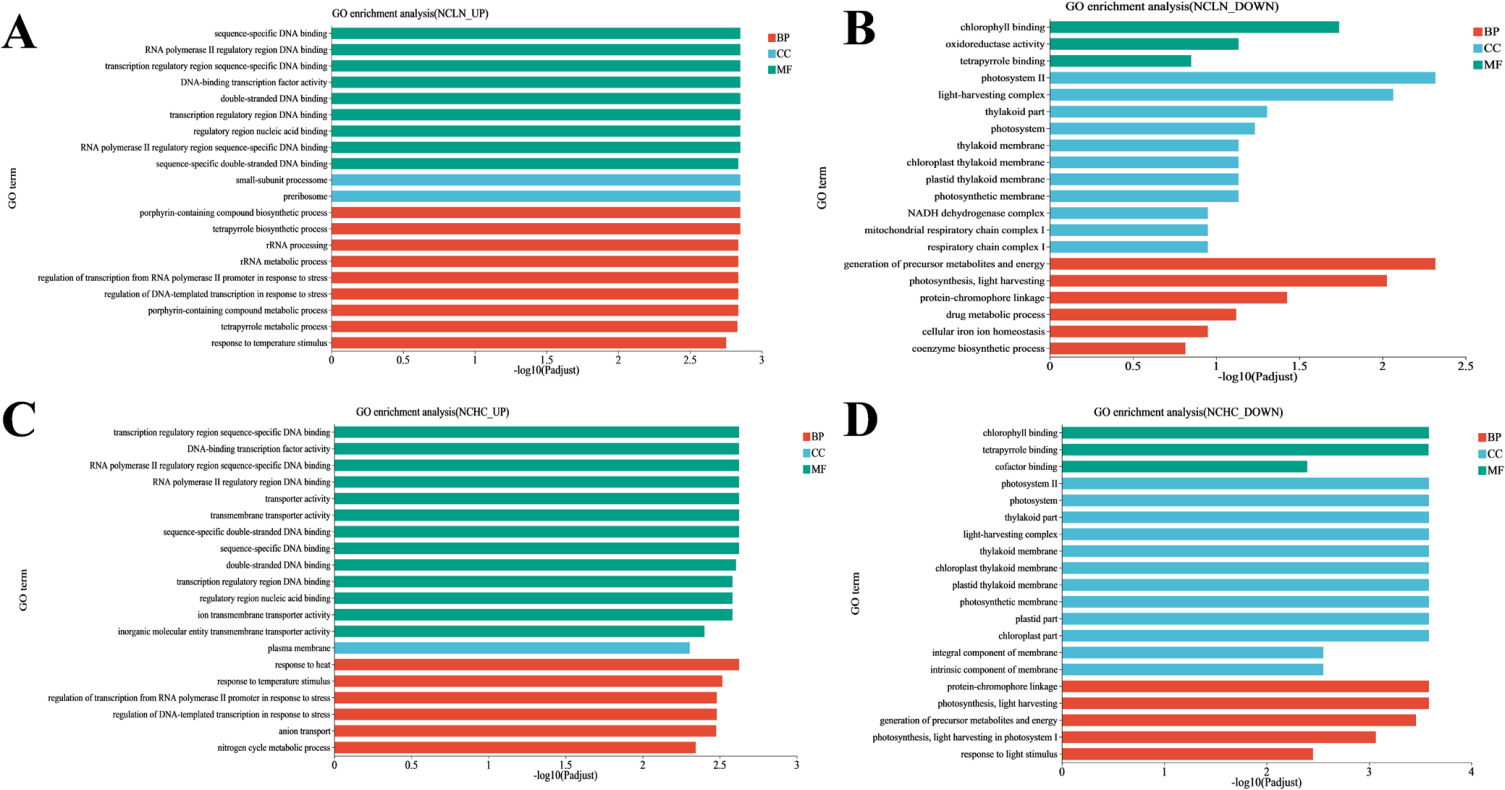
Enriched GO pathways analysis of differentially expressed genes (all *p* < 0.05). (A) Upregulated genes in LN/NC. (B) Downregulated genes in LN/NC. (C) Upregulated genes in HC/NC. (D) Downregulated genes in HC/NC. Three colors represent three major categories, namely biological processes (BP), cellular components (CC), and molecular functions (MF).

Volcano plots illustrate the distribution of DEGs between the LN/NC and HC/NC groups (Figure 2A,B). In the NC versus LN comparison, highly significant up‐regulated genes included several integral membrane proteins (e.g., *PHATRDRAFT_49182*) and a gene involved in porphyrin biosynthesis (*PHATRDRAFT_20757*). Strong fold increases were also observed for a potassium ion leak channel (*PHATRDRAFT_46431*). On the down‐regulated side, iron starvation‐induced proteins (e.g., *PHATRDRAFT_54465*, *PHATRDRAFT_54987*) were most prominent, indicating shifts in membrane transport and iron metabolism under nitrogen limitation (Figure 2A). In the NC versus HC comparison, significantly up‐regulated genes included those associated with fatty acid biosynthesis and redox processes (*PHATRDRAFT_35939*), as well as a potassium ion leak channel (*PHATRDRAFT_46431*). Down‐regulated genes were enriched in energy metabolism (e.g., *PHATRDRAFT_23658*) and iron starvation‐induced proteins (e.g., *PHATRDRAFT_54987*), suggesting adjustments in lipid metabolism, ion transport, and energy production under elevated CO_2_ (Figure 2B).

In the clustered heatmaps of the top 50 DEGs, several genes associated with carbon and nitrogen metabolism were clearly differentiated under low nitrogen and high CO_2_ treatments (Figure 2E,F). For carbon metabolism, gene‐PHATRDRAFT_35164 (phosphoglycerate mutase, PGAM_7), a key enzyme in glycolysis/gluconeogenesis, showed consistently high expression in low nitrogen conditions, whereas in high CO_2_ expression was variable. For nitrogen metabolism, gene‐PHATRDRAFT_13154 and PHATRDRAFT_8155 (encoding nitrite reductase, NirB), PHATRDRAFT_54983 (nitrate reductase, NR), and PHATRDRAFT_26029 (nitrate transporter, NRTs) were all strongly upregulated under low nitrogen conditions, while their expression patterns were less consistent under high CO_2_ stress. These results suggest that nitrogen assimilation pathways were more strongly induced under low nitrogen than high CO_2_ conditions, while glycolytic activity was also enhanced under nitrogen limitation.

**FIGURE 4:**
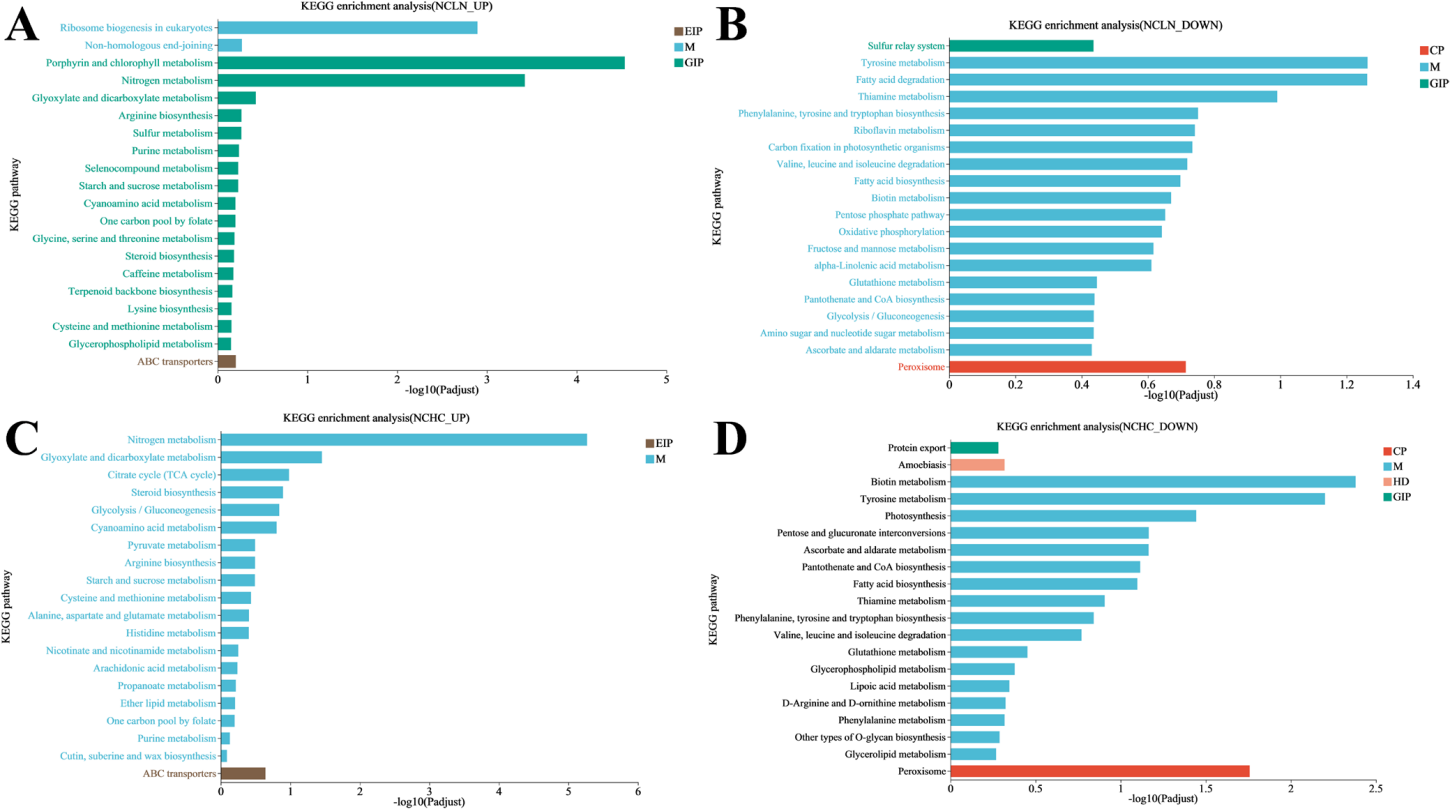
Enriched KEGG pathways analysis of differentially expressed genes (all *p* < 0.05). (A) Upregulated genes in LN/NC. (B) Downregulated genes in LN/NC. (C) Upregulated genes in HC/NC. (D) Downregulated genes in HC/NC. Different colors represent the seven branches of the KEGG metabolic pathway, namely metabolism (M), genetic information processing (GIP), environmental information processing (EIP), cellular processes (CP), human diseases (HD).

#### 3.3.2 Functional Enrichment Analysis of DEGs

In the normal conditions and low nitrogen conditions, upregulated genes were significantly enriched in processes related to tetrapyrrole and porphyrin biosynthesis, rRNA metabolic processes, and stress‐responsive transcriptional regulation, reflecting enhanced nitrogen utilization and stress adaptation. Enrichment in the small‐subunit processome and preribosome further suggested increased ribosome biogenesis (Figure 3A). In contrast, downregulated genes were mainly enriched in photosynthesis and light‐harvesting processes, with associated cellular components such as photosystem II and thylakoid membrane, consistent with a reduction in photosynthetic activity under nitrogen limitation (Figure 3B). The results of the KEGG analysis revealed significant enrichment of differentially upregulated genes under low nitrogen conditions in pathways involving porphyrin and chlorophyll metabolism, consistent with previous reports that *P. tricornutum* components rich in N are degraded to provide the nitrogen necessary for certain processes. In addition, nitrogen metabolism was also upregulated. The glyoxylate and dicarboxylate metabolism pathways were also upregulated (Figure 4A). Differentially downregulated genes under low nitrogen conditions were significantly enriched in pathways that involved tyrosine metabolism, the pentose phosphate pathway, carbon fixation in photosynthetic organisms, lipid metabolism, and the metabolism of cofactors and vitamins, indicating a decrease in cell activities (Figure 4B).

**FIGURE 5:**
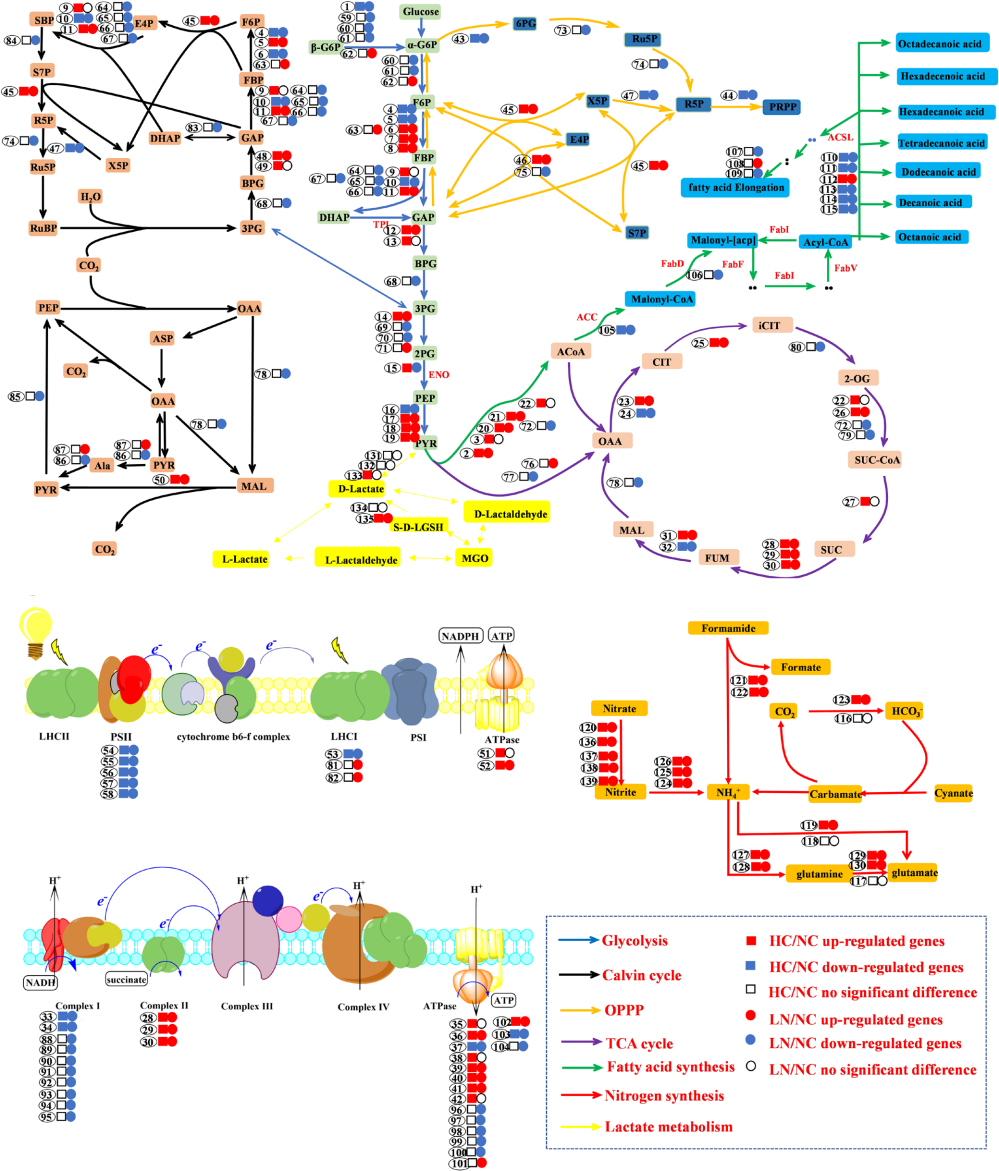
Differentially expressed genes involved in metabolic pathways under LN and HC conditions. The numbers represent the gene numbers listed in Supplementary Table S6, and complete information about the genes can be obtained. Squares represent comparisons between HC and NC, while circles represent comparisons between LN and NC. Colors indicate differential expression: red = upregulated genes, green = downregulated genes, and white = no significant difference. Arrows represent the direction of metabolic flux, and different arrow colors correspond to distinct metabolic pathways.

In the normal conditions and high CO_2_ conditions, upregulated genes were enriched in transcription factor activity, transmembrane transporter activity, and stress response pathways, suggesting transcriptional reprogramming and enhanced environmental responsiveness (Figure 3C). Conversely, downregulated genes were enriched in photosynthetic processes and components such as photosystem II and light‐harvesting complexes, indicating suppression of photosynthetic machinery under elevated CO_2_ conditions (Figure 3D). Differentially upregulated genes under high CO_2_ conditions were significantly enriched in pathways including arginine metabolism, glyoxylate and dicarboxylate metabolism, citrate cycle, glycolysis/gluconeogenesis, pyruvate metabolism, starch and sucrose metabolism, carbon fixation in photosynthetic organisms, steroid biosynthesis, nitrogen metabolism, ABC transporters, and cyanoamino acid metabolism, indicating an increase in cell activities (Figure 4C). Differentially downregulated genes under high CO_2_ conditions were significantly enriched in pathways including tyrosine metabolism, pentose and glucuronate interconversions, ascorbate and aldarate metabolism, photosynthesis, amoebiasis, fatty acid biosynthesis, and glycerophospholipid metabolism (Figure 4D).

#### 3.3.3 Carbon Metabolism Analysis

Carbon metabolism is central to the growth and energy supply of microalgae, influencing processes such as glycolysis, the TCA cycle, and carbon fixation. Under high carbon availability, *P. tricornutum* may activate or adjust various carbon metabolic pathways to efficiently utilize excess carbon sources while maintaining redox and energy balance. To explore how *P. tricornutum* modulates carbon metabolism under high‐carbon and low‐nitrogen conditions, we analyzed the expression profiles of genes involved in key carbon metabolic pathways.

PGAM_7 (phosphoglycerate mutase), involved in the glycolysis/gluconeogenesis pathway, was significantly upregulated with log_2_FCs of 7.56 and 7.46 in both LN/NC and HC/NC groups (Table S6), suggesting an enhanced glycolytic process to meet energy demands. PHATRDRAFT_40430 (dihydrolipoamide succinyltransferase) showed significant upregulation with log_2_FCs of 3.84 and 4.62 in the LN/NC and HC/NC groups, respectively (Table S6), indicative of an accelerated TCA cycle to support energy production. FBPC4 (fructose‐1,6‐bisphosphatase), which is involved in the glycolysis/gluconeogenesis pathway, the pentose phosphate pathway, and the carbon fixation pathway in photosynthetic organisms, was significantly downregulated with log_2_FCs of 7.57 and 2.10 respectively in the LN/NC and HC/NC groups (Table S6). FBPC4 potentially adapts metabolic flux to environmental changes.

#### 3.3.4 Nitrogen Metabolism Analysis

Nitrogen is an essential nutrient for microalgal growth, as it is required for the synthesis of amino acids, nucleotides, and other nitrogen‐containing biomolecules. Under low nitrogen conditions, microalgae often undergo extensive metabolic reprogramming to optimize nitrogen assimilation and conserve nitrogen usage. In *P. tricornutum* , nitrogen metabolism involves key processes such as nitrate and nitrite reduction, ammonium assimilation, and amino acid biosynthesis. To investigate how *P. tricornutum* responds to low nitrogen and high CO_2_ conditions, we analyzed the expression of genes involved in major nitrogen metabolic pathways.

In the nitrogen metabolism pathway, PHATRDRAFT_13154 and PHATRDRAFT_8155 (encoding nitrite reductase, NirB), with PHATRDRAFT_27757 (ferredoxin–nitrite reductase, NirA) are involved in converting nitrite to ammonium. PHATRDRAFT_13154 was the most upregulated gene in both the LN/NC and HC/NC groups (Figure 5), with log_2_FCs of 12.00 and 11.31, respectively (Table S6). PHATRDRAFT_54983 (nitrate reductase, NR) was upregulated with log_2_FCs of 7.41 in the LN/NC group and 6.29 in the HC/NC group (Table S6). It catalyzes the conversion of nitrate to nitrite, suggesting optimized nitrogen metabolism gene expression to improve resource utilization efficiency. In addition to nitrate reductase, several genes encoding nitrate transporters (NRTs) were consistently upregulated under both LN/NC and HC/NC conditions, including PHATRDRAFT_54560, PHATRDRAFT_40691, PHATRDRAFT_26029, and PHATRDRAFT_2032 (Table S6). Their expression changes ranged from moderate (log_2_FC ~1.2–2.8) to strong induction (log_2_FC > 5), suggesting that *P. tricornutum* may enhance nitrate uptake capacity when nitrogen availability is limited or when carbon supply is elevated. This coordinated upregulation of nitrate transporters and nitrate reductase highlights a strategy to optimize nitrogen acquisition and maintain intracellular nitrogen homeostasis under stress conditions.

#### 3.3.5 Lactic Acid Metabolism Analysis

Lactic acid metabolism is implicated in cellular energy homeostasis, particularly under stress conditions where metabolic fluxes are altered. In microalgae such as *P. tricornutum* , shifts in carbon and nitrogen availability may disrupt the balance of key cofactors like NADH/NAD^+^, prompting the activation of alternative metabolic pathways such as lactate production; this reaction regenerates NAD^+^ to sustain basal glycolytic ATP synthesis and thus contributes to cellular energy homeostasis under carbon‐excess, nitrogen‐limiting conditions (Alipanah et al. 2015; Levitan et al. 2015; Yang et al. 2013). Given the high carbon and low nitrogen conditions applied in this study, we examined the expression of genes involved in lactic acid metabolism to better understand how *P. tricornutum* adjusts its energy metabolism in response to nutrient stress.

PHATRDRAFT_43667 (D‐lactate dehydrogenase), involved in lactate metabolism, exhibited significant upregulation in both the LN/NC and HC/NC groups (Figure 5), indicating potential metabolic adaptations to different environmental conditions. PHATRDRAFT_bd1469 (L‐lactate dehydrogenase, LDHA) showed minimal expression in the LN/NC but was upregulated in the HC/NC, likely due to the heightened energy demands under high CO_2_ conditions.

### 3.4 Comparison of qPCR and RNA‐Seq Results

To verify the results of RNA sequencing, we perform qPCR with RPS and 18S as internal references. Analysis on GOX, PGP_2, gloB, NRT, formidase, bglX, petJ, and yjgB was performed. The results of target genes detected by qPCR were consistent with the RNA sequence analysis (Figure S1), which indicated that our transcriptim results‐based RNA sequencing was highly reliable and reproducible.

## 4 Discussion

Diatoms thrive in dynamic marine environments where fluctuations in carbon and nitrogen availability frequently disrupt metabolic homeostasis. This study highlights how *P. tricornutum* responds to such environmental stress primarily through mechanisms related to carbon–nitrogen (C—N) imbalance. Both high CO_2_ and low nitrogen treatments imposed distinct but converging pressures on cellular metabolism, driving a global metabolic reprogramming centred on maintaining the balance between carbon assimilation and nitrogen utilization. Such C—N imbalance represents a fundamental challenge for phytoplankton acclimation in future ocean conditions (Flynn et al. 2010; Bach and Taucher 2019; Bayramova et al. 2023).

Our transcriptomic data revealed that multiple central metabolic pathways, including glycolysis, the TCA cycle, and oxidative phosphorylation, were activated under both conditions. The upregulation of phosphoglycerate mutase (PGAM_7) and dihydrolipoamide succinyltransferase suggests enhanced carbon flux through energy‐generating pathways, consistent with the need to provide carbon skeletons and ATP for nitrogen assimilation under nutrient stress. These findings echo previous reports that diatoms boost central carbon metabolism to compensate for limited nitrogen supply (Levitan et al. 2015; Remmers et al. 2018). Similar metabolic adjustments have also been reported in *Thalassiosira pseudonana* under combined light and nitrogen stress (Li et al. 2023), suggesting a conserved metabolic strategy among diatoms to maintain C—N balance under fluctuating environments.

Concurrently, the pronounced upregulation of nitrogen metabolism genes, particularly nitrite reductases (PHATRDRAFT_13154, PHATRDRAFT_8155) and ferredoxin–nitrite reductase (PHATRDRAFT_27757), underscores the primary cellular objective of maximizing nitrogen utilization efficiency. This finding aligns with previous studies showing that diatoms prioritize nitrogen scavenging and assimilation under deficiency (Burrows et al. 2012). The strong induction of nitrite reductase (PHATRDRAFT_13154) (log_2_FC ~12 in LN/NC) is consistent with the transcriptional reprogramming reported by Scarsini et al. (2022) during nitrogen deprivation. Moreover, the upregulation of nitrate reductase (PHATRDRAFT_54983) under high CO_2_ may represent a mechanism to optimize the C—N ratio by enhancing nitrogen assimilation when carbon is abundant, thereby preventing a severe internal C—N imbalance. However, compared with *Thalassiosira oceanica*, which tends to downregulate nitrate transporter genes under prolonged nitrogen depletion (Li et al. 2021), *P. tricornutum* appears to maintain active nitrate reduction, suggesting species‐specific nitrogen acquisition strategies.

The adjustments in lactate metabolism (e.g., D‐ and L‐lactate dehydrogenases) further reflect the metabolic flexibility of *P. tricornutum* in managing redox balance and carbon flow under different C—N regimes. This adaptability is consistent with the concept of metabolic reprogramming where secondary pathways are modulated to support core metabolic homeostasis under stress (Levitan et al. 2015). Moreover, our enrichment results indicate activation of lactic acid fermentation and nitrogen salvage pathways, pointing toward an energy‐conserving, nutrient‐recycling strategy under stress. Similar responses have been observed in *Thalassiosira* species under combined light and nitrogen perturbations (Ma et al. 2024) and other multi‐stress conditions (Li et al. 2023), suggesting that certain stress‐responsive modules are conserved across diatoms, despite differences in their ecological niches.

In summary, our results delineate an integrated metabolic response in *P. tricornutum* to C—N imbalance, characterized by the synergistic enhancement of carbon processing and nitrogen assimilation pathways. While this study provides a transcriptomic overview, integrating proteomic and metabolomic data would further clarify post‐transcriptional regulation. Furthermore, functional characterization of highly induced genes via genetic manipulation could reveal their specific roles in diatom acclimation. These findings advance understanding of diatom metabolic plasticity under future ocean scenarios and highlight potential genetic targets for enhancing strain performance in biotechnological and carbon sequestration applications.

## 5 Conclusions

This study offers an in‐depth understanding of *P. tricornutum's* metabolic regulatory mechanisms under different carbon and nitrogen conditions. The findings highlight key genes and pathways responsive to carbon and nitrogen conditions, suggesting how *P. tricornutum* modulates its transcriptional program under environmental changes. These results provide valuable information for understanding diatom responses to ocean change and may inform future biotechnological applications.

## Author Contributions

**Yi Zhang:** formal analysis (equal), methodology (equal), writing – original draft (equal), writing – review and editing (equal). **Jiawen Duan:** conceptualization (equal), data curation (equal), formal analysis (equal). **Yimeng Zheng:** investigation (equal), methodology (equal). **Xiaoqi Chen:** resources (equal), software (equal). **Chenhui Li:** data curation (equal). **Zhenyu Xie:** conceptualization (equal), project administration (equal), supervision (equal), writing – review and editing (equal). **Aiyou Huang:** conceptualization (equal), project administration (equal), supervision (equal), validation (equal), writing – review and editing (equal).

## Conflicts of Interest

The authors declare no conflicts of interest.

## Supporting information


**Table S1:** Primer sequences for randomly selected transcriptome‐derived genes.

**Table S2:** Quality assessment table of RNA‐seq data.

**Table S3:** Statistics of reads ratio mapping to the reference genome.

**Table S4:** Differentially expressed genes under LN.

**Table S5:** Differentially expressed genes under HC.

**Table S6:** Differentially expressed genes involved in specific metabolic pathways in the NC _ versus _ HC and NC _ versus _ LN experimental groups.

**Figure S1:** Comparison of gene expression levels determined by RNA‐seq and qPCR: (A) LN/NC; (B) HC/NC. Gene abbreviations: bglX, beta‐glucosidase; DAO, predicted protein; glnA, glutamine synthase; gloB, predicted protein; GOX, glycolate oxidase; lctP, LCTP l‐lactate permease; NRT, predicted protein; petJ, cytochrome c6; PGP_2, phosphoglycolate phosphatase; SHMT, serine hydroxymethyltransferase; yjgB, predicted protein. Expression levels were normalized to RPS and 18S reference genes and calculated using the 2^−ΔΔCt^ method.

## Data Availability

The raw data of RNA‐Seq have been deposited into the National Center for Biotechnology Information (NCBI) SRA database with the BioProject number of PRJNA1289763. The data are publicly available at: https://www.ncbi.nlm.nih.gov/bioproject/PRJNA1289763.

## References

[ece372754-bib-0001] Alipanah, L. , J. Rohloff , P. Winge , A. M. Bones , and T. Brembu . 2015. “Whole‐Cell Response to Nitrogen Deprivation in the Diatom *Phaeodactylum tricornutum* .” Journal of Experimental Botany 66, no. 20: 6281–6296. 26163699 10.1093/jxb/erv340 PMC4588885

[ece372754-bib-0002] Bach, L. T. , and J. Taucher . 2019. “CO_2_ Effects on Diatoms: A Synthesis of More Than a Decade of Ocean Acidification Experiments With Natural Communities.” Ocean Science 15, no. 4: 1159–1175.

[ece372754-bib-0003] Bayramova, E. M. , Y. D. Bedoshvili , and Y. V. Likhoshway . 2023. “Molecular and Cellular Mechanisms of Diatom Response to Environmental Changes.” Limnology and Freshwater Biology 1: 20–30.

[ece372754-bib-0004] Bowler, C. , A. E. Allen , J. H. Badger , et al. 2008. “The *Phaeodactylum* Genome Reveals the Evolutionary History of Diatom Genomes.” Nature 456, no. 7219: 239–244. 18923393 10.1038/nature07410

[ece372754-bib-0005] Burrows, E. H. , N. B. Bennette , D. Carrieri , et al. 2012. “Dynamics of Lipid Biosynthesis and Redistribution in the Marine Diatom *Phaeodactylum tricornutum* Under Nitrate Deprivation.” Bioenergy Research 5: 876–885.

[ece372754-bib-0006] Conesa, A. , P. Madrigal , S. Tarazona , et al. 2016. “A Survey of Best Practices for RNA‐Seq Data Analysis.” Genome Biology 17: 1–19. 26813401 10.1186/s13059-016-0881-8 PMC4728800

[ece372754-bib-0007] De Risco, V. , R. Raniello , F. Maumus , et al. 2009. “Gene Silencing in the Marine Diatom Phaeodactylum Tricornutum.” Nucleic Acids Research 37, no. 14: e96. 19487243 10.1093/nar/gkp448 PMC2724275

[ece372754-bib-0008] Feely, R. A. , L. Q. Jiang , R. Wanninkhof , et al. 2023. “Acidification of the Global Surface Ocean.” Oceanography 36: 120–129.

[ece372754-bib-0009] Feely, R. A. , R. R. Okazaki , W. J. Cai , et al. 2018. “The Combined Effects of Acidification and Hypoxia on pH and Aragonite Saturation in the Coastal Waters of the California Current Ecosystem and the Northern Gulf of Mexico.” Continental Shelf Research 152: 50–60.

[ece372754-bib-0010] Flynn, K. J. , J. A. Raven , T. A. V. Rees , et al. 2010. “Is the Growth Rate Hypothesis Applicable to Microalgae?” Journal of Phycology 46, no. 1: 1–12.

[ece372754-bib-0011] Gruber, N. 2008. “The Marine Nitrogen Cycle: Overview and Challenges.” Nitrogen in the Marine Environment 2: 1–50.

[ece372754-bib-0012] Guillard, R. R. L. 1975. “Culture of Phytoplankton for Feeding Marine Invertebrates.” In Culture of Marine Invertebrate Animals, edited by W. L. Smith and M. H. Chanley , 29–60. Springer.

[ece372754-bib-0013] Hu, Q. , M. Sommerfeld , E. Jarvis , et al. 2008. “Microalgal Triacylglycerols as Feedstocks for Biofuel Production: Perspectives and Advances.” Plant Journal 54, no. 4: 621–639. 10.1111/j.1365-313X.2008.03492.x 18476868

[ece372754-bib-0014] Huang, A. , S. Wu , W. Gu , Y. Li , X. Xie , and G. Wang . 2019. “Provision of Carbon Skeleton for Lipid Synthesis From the Breakdown of Intracellular Protein and Soluble Sugar in *Phaeodactylum tricornutum* Under High CO_2_ .” BMC Biotechnology 19, no. 1: 53. 31349823 10.1186/s12896-019-0544-4 PMC6659225

[ece372754-bib-0015] John, J. S. 2013. “SeqPrep (v1.2).” https://github.com/jstjohn/SeqPrep.

[ece372754-bib-0016] Joshi, N. A. , and J. N. Fass . 2011. “Sickle: A Sliding‐Window, Adaptive, Quality‐Based Trimming Tool for FastQ Files (v1.33).” https://github.com/najoshi/sickle.

[ece372754-bib-0017] Klopfenstein, D. V. , L. Zhang , B. S. Pedersen , et al. 2018. “GOATOOLS: A Python Library for Gene Ontology Analyses.” Scientific Reports 8, no. 1: 10872. 30022098 10.1038/s41598-018-28948-z PMC6052049

[ece372754-bib-0018] Levitan, O. , J. Dinamarca , E. Zelzion , et al. 2015. “Remodeling of Intermediate Metabolism in the Diatom *Phaeodactylum tricornutum* Under Nitrogen Stress.” Proceedings of the National Academy of Sciences of the United States of America 112, no. 2: 412–417. 25548193 10.1073/pnas.1419818112 PMC4299248

[ece372754-bib-0019] Li, B. , and C. N. Dewey . 2011. “RSEM: Accurate Transcript Quantification From RNA‐Seq Data With or Without a Reference Genome.” BMC Bioinformatics 12: 323. 21816040 10.1186/1471-2105-12-323 PMC3163565

[ece372754-bib-0020] Li, W. , K. Gao , and J. Beardall . 2012. “Interactive Effects of Ocean Acidification and Nitrogen‐Limitation on the Diatom *Phaeodactylum tricornutum* .” PLoS One 7, no. 12: e51590. 23236517 10.1371/journal.pone.0051590 PMC3517544

[ece372754-bib-0021] Li, Z. , W. Li , Y. Zhang , et al. 2021. “Dynamic Photophysiological Stress Response of a Model Diatom to Ten Environmental Stresses.” Journal of Phycology 57, no. 2: 484–495. 32945529 10.1111/jpy.13072

[ece372754-bib-0022] Li, Z. , Y. Zhang , W. Li , A. J. Irwin , and Z. V. Finkel . 2023. “Common Environmental Stress Responses in a Model Marine Diatom.” New Phytologist 240, no. 1: 272–284. 37488721 10.1111/nph.19147

[ece372754-bib-0023] Livak, K. J. , and T. D. Schmittgen . 2001. “Analysis of Relative Gene Expression Data Using Real‐Time Quantitative PCR and the 2−ΔΔCT Method.” Methods 25, no. 4: 402–408. 11846609 10.1006/meth.2001.1262

[ece372754-bib-0024] Ma, X. , Z. Qin , K. B. Johnson , et al. 2024. “Transcriptomic Responses to Shifts in Light and Nitrogen in Two Congeneric Diatom Species.” Frontiers in Microbiology 15: 1437274. 39206371 10.3389/fmicb.2024.1437274 PMC11349689

[ece372754-bib-0025] Nelson, D. M. , P. Treguer , M. A. Brzezinski , A. Leynaert , and B. Quéguiner . 1995. “Production and Dissolution of Biogenic Silica in the Ocean: Revised Global Estimates, Comparison With Regional Data and Relationship to Biogenic Sedimentation.” Global Biogeochemical Cycles 9, no. 3: 359–372.

[ece372754-bib-0026] Pelusi, A. , L. Ambrosino , M. Miralto , et al. 2023. “Gene Expression During the Formation of Resting Spores Induced by Nitrogen Starvation in the Marine Diatom *Chaetoceros socialis* .” BMC Genomics 24, no. 1: 106. 36899305 10.1186/s12864-023-09175-x PMC9999646

[ece372754-bib-0027] Raven, J. A. , and A. M. Waite . 2004. “The Evolution of Silicification in Diatoms: Inescapable Sinking and Sinking as Escape?” New Phytologist 162, no. 1: 45–61.

[ece372754-bib-0028] Remmers, I. M. , S. D'Adamo , D. E. Martens , et al. 2018. “Orchestration of Transcriptome, Proteome and Metabolome in the Diatom *Phaeodactylum tricornutum* During Nitrogen Limitation.” Algal Research 35: 33–49.

[ece372754-bib-0029] Robinson, M. D. , D. J. McCarthy , and G. K. Smyth . 2010. “edgeR: A Bioconductor Package for Differential Expression Analysis of Digital Gene Expression Data.” Bioinformatics 26: 139–140. 19910308 10.1093/bioinformatics/btp616 PMC2796818

[ece372754-bib-0030] Scarsini, M. , S. Thiriet‐Rupert , B. Veidl , et al. 2022. “The Transition Toward Nitrogen Deprivation in Diatoms Requires Chloroplast Stand‐By and Deep Metabolic Reshuffling.” Frontiers in Plant Science 12: 760516. 35126407 10.3389/fpls.2021.760516 PMC8811913

[ece372754-bib-0031] Shimakawa, G. , M. Demulder , S. Flori , et al. 2024. “Diatom Pyrenoids Are Encased in a Protein Shell That Enables Efficient CO_2_ Fixation.” Cell 187, no. 21: 5919–5934.e19. 39357521 10.1016/j.cell.2024.09.013

[ece372754-bib-0032] Siaut, M. , M. Heijde , M. Mangogna , et al. 2007. “Molecular Toolbox for Studying Diatom Biology in *Phaeodactylum tricornutum* .” Gene 406, no. 1–2: 23–35. 17658702 10.1016/j.gene.2007.05.022

[ece372754-bib-0033] Stukenberg, D. , S. Zauner , G. Dell'Aquila , et al. 2018. “Optimizing CRISPR/Cas9 for the Diatom *Phaeodactylum tricornutum* .” Frontiers in Plant Science 9: 740. 29928285 10.3389/fpls.2018.00740 PMC5998643

[ece372754-bib-0034] Sunda, W. G. , and W. J. Cai . 2012. “Eutrophication Induced CO_2_‐Acidification of Subsurface Coastal Waters: Interactive Effects of Temperature, Salinity, and Atmospheric pCO_2_ .” Environmental Science & Technology 46, no. 19: 10651–10659. 22889106 10.1021/es300626f

[ece372754-bib-0035] Trapnell, C. , L. Pachter , and S. L. Salzberg . 2009. “TopHat: Discovering Splice Junctions With RNA‐Seq.” Bioinformatics 25: 1105–1111. 19289445 10.1093/bioinformatics/btp120 PMC2672628

[ece372754-bib-0036] Wang, W. , H. Fang , M. Aslam , et al. 2022. “MYB Gene Family in the Diatom *Phaeodactylum tricornutum* Revealing Their Potential Functions in the Adaption to Nitrogen Deficiency and Diurnal Cycle.” Journal of Phycology 58, no. 1: 121–132. 34634129 10.1111/jpy.13217

[ece372754-bib-0037] Wu, S. , W. Gu , A. Huang , et al. 2019. “Elevated CO_2_ Improves Both Lipid Accumulation and Growth Rate in the Glucose‐6‐Phosphate Dehydrogenase Engineered *Phaeodactylum tricornutum* .” Microbial Cell Factories 18, no. 1: 161. 31547820 10.1186/s12934-019-1214-x PMC6757359

[ece372754-bib-0038] Wu, S. , A. Huang , B. Zhang , et al. 2015. “Enzyme Activity Highlights the Importance of the Oxidative Pentose Phosphate Pathway in Lipid Accumulation and Growth of *Phaeodactylum tricornutum* Under CO_2_ Concentration.” Biotechnology for Biofuels 8, no. 1: 78. 26052345 10.1186/s13068-015-0262-7 PMC4456714

[ece372754-bib-0039] Wu, Y. , K. Gao , and U. Riebesell . 2010. “CO_2_‐Induced Seawater Acidification Affects Physiological Performance of the Marine Diatom *Phaeodactylum tricornutum* .” Biogeosciences 7, no. 9: 2915–2923.

[ece372754-bib-0040] Xie, C. , X. Mao , J. Huang , et al. 2011. “KOBAS 2.0: A Web Server for Annotation and Identification of Enriched Pathways and Diseases.” Nucleic Acids Research 39: W316–W322. 21715386 10.1093/nar/gkr483 PMC3125809

[ece372754-bib-0041] Xu, D. , C. E. Schaum , B. Li , et al. 2021. “Acclimation and Adaptation to Elevated pCO_2_ Increase Arsenic Resilience in Marine Diatoms.” ISME Journal 15, no. 6: 1599–1613. 33452476 10.1038/s41396-020-00873-y PMC8163839

[ece372754-bib-0042] Yang, Z. K. , Y. H. Ma , J. W. Zheng , W. D. Yang , J. S. Liu , and H. Y. Li . 2014. “Proteomics to Reveal Metabolic Network Shifts Towards Lipid Accumulation Following Nitrogen Deprivation in the Diatom *Phaeodactylum tricornutum* .” Journal of Applied Phycology 26: 73–82. 24600163 10.1007/s10811-013-0050-3 PMC3918386

[ece372754-bib-0043] Yang, Z. K. , Y. F. Niu , Y. H. Ma , et al. 2013. “Molecular and Cellular Mechanisms of Neutral Lipid Accumulation in Diatom Following Nitrogen Deprivation.” Biotechnology for Biofuels 6: 1–14. 23642220 10.1186/1754-6834-6-67 PMC3662598

[ece372754-bib-0044] Zaslavskaia, L. A. , J. C. Lippmeier , P. G. Kroth , A. R. Grossman , and K. E. Apt . 2000. “Transformation of the Diatom *Phaeodactylum tricornutum* (Bacillariophyceae) With a Variety of Selectable Marker and Reporter Genes.” Journal of Phycology 36, no. 2: 379–386.

