# Supplementary material for: Transcriptomic Responses of the Marine Diatom Phaeodactylum tricornutum to High Carbon and Low Nitrogen Stress
Source: Ecol Evol. 2026 Jan 9;16(1):e72754. doi: 10.1002/ece3.72754 (PMC12789813; doi:10.1002/ece3.72754)
Supplement: Supplementary file 7 — Figure S1: Comparison of gene expression levels determined by RNA‐seq and qPCR: (A) LN/NC; (B) HC/NC. Gene abbreviations: bglX, beta‐glucosidase; DAO, predicted protein; glnA, glutamine synthase; gloB, predicted protein; GOX, glycolate oxidase; lctP, LCTP l‐lactate permease; NRT, predicted protein; petJ, cytochrome c6; PGP_2, phosphoglycolate phosphatase; SHMT, serine hydroxymethyltransferase; yjgB, predicted protein. Expression levels were normalized to RPS and 18S reference genes and calculated using the 2−ΔΔCt method. [file ECE3-16-e72754-s001.docx]

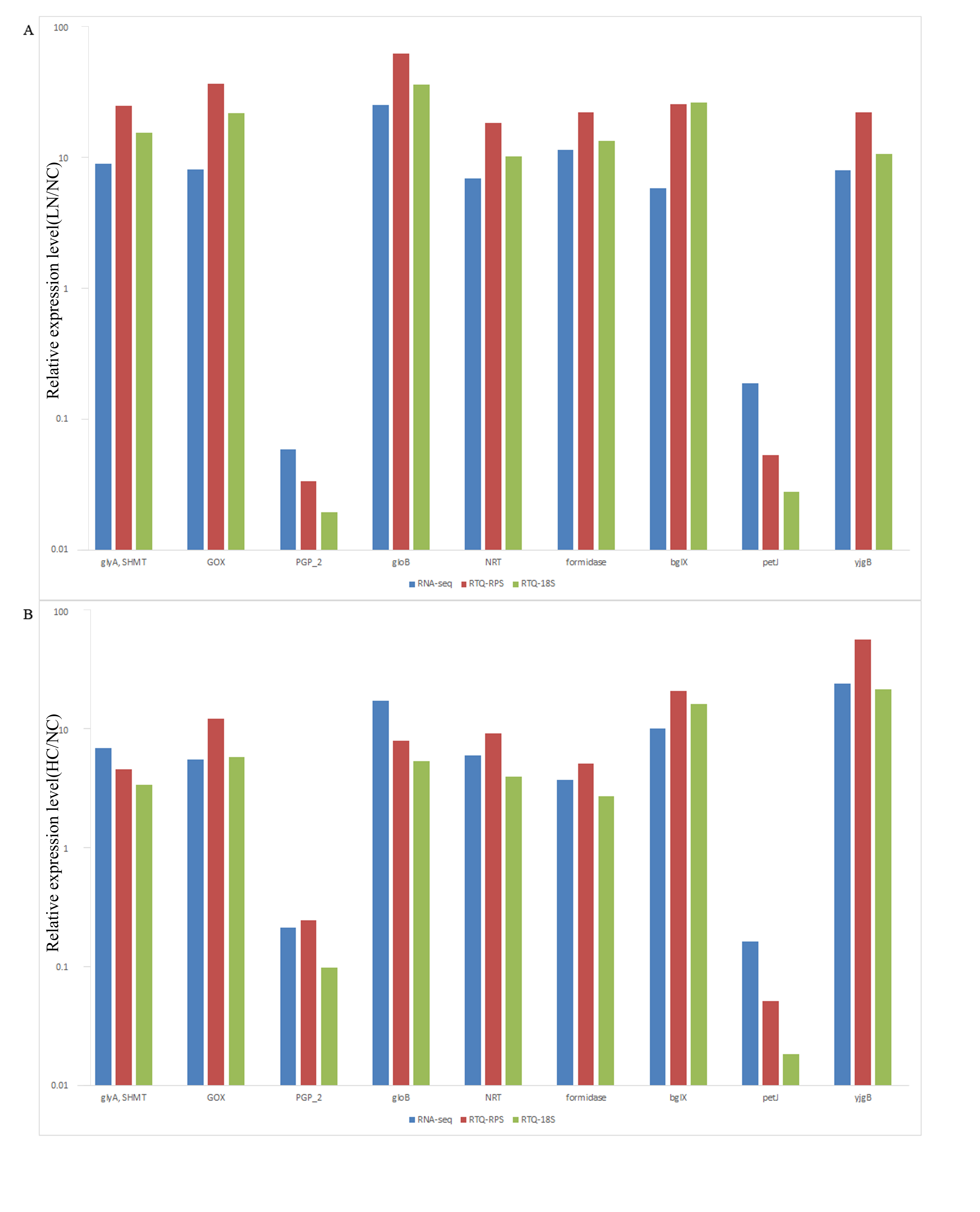


Figure S1. Comparison of gene expression levels determined by RNA-seq and qPCR. (A). LN/NC. (B) HC/NC. Gene abbreviations: SHMT, serine hydroxymethyltransferase; GOX, glycolate oxidase; PGP_2, phosphoglycolate phosphatase, gloB, predicted protein; NRT, predicted protein; bglX, beta-glucosidase; lctP, LCTP l-lactate permease; DAO, predicted protein, glnA, glutamine synthase; petJ, cytochrome c6; yjgB, predicted protein. Expression levels were normalized to RPS and 18S reference genes and calculated using the 2^-ΔΔCt method.
